# Leveraging family history data to disentangle time-varying effects on disease risk using lifecourse mendelian randomization

**DOI:** 10.1007/s10654-023-01001-8

**Published:** 2023-05-08

**Authors:** Tom G Richardson, Helena Urquijo, Michael V Holmes, George Davey Smith

**Affiliations:** MRC Integrative Epidemiology Unit (IEU), Population Health Sciences, Bristol Medical School, University of Bristol, Oakfield House, Oakfield Grove, Bristol, BS8 2BN UK

**Keywords:** Childhood body size, Mendelian randomization, Lifecourse epidemiology, Family disease history, UK Biobank

## Abstract

Lifecourse Mendelian randomization is a causal inference technique which harnesses genetic variants with time-varying effects to develop insight into the influence of age-dependent lifestyle factors on disease risk. Here, we apply this approach to evaluate whether childhood body size has a direct consequence on 8 major disease endpoints by analysing parental history data from the UK Biobank study.

Our findings suggest that, whilst childhood body size increases later risk of outcomes such as heart disease (odds ratio (OR) = 1.15, 95% CI = 1.07 to 1.23, P = 7.8 × 10^− 5^) and diabetes (OR = 1.43, 95% CI = 1.31 to 1.56, P = 9.4 × 10^− 15^) based on parental history data, these findings are likely attributed to a sustained influence of being overweight for many years over the lifecourse. Likewise, we found evidence that remaining overweight throughout the lifecourse increases risk of lung cancer, which was partially mediated by lifetime smoking index. In contrast, using parental history data provided evidence that being overweight in childhood may have a protective effect on risk of breast cancer (OR = 0.87, 95% CI = 0.78 to 0.97, P = 0.01), corroborating findings from observational studies and large-scale genetic consortia.

Large-scale family disease history data can provide a complementary source of evidence for epidemiological studies to exploit, particularly given that they are likely more robust to sources of selection bias (e.g. survival bias) compared to conventional case control studies. Leveraging these data using approaches such as lifecourse Mendelian randomization can help elucidate additional layers of evidence to dissect age-dependent effects on disease risk.

**Supplementary Information:**

The online version contains supplementary material available at 10.1007/s10654-023-01001-8.

## Introduction

Disentangling causal from correlated risk factors which can vary over the lifecourse is a challenging and arduous task in a conventional epidemiological setting. Overcoming these obstacles is central to the conception and implementation of an approach known as Mendelian randomization (MR), a causal inference method which harnesses genetic variants as instrumental variables to estimate the effect of risk factors on disease outcomes [1, 2]. MR exploits the properties of naturally occurring genetic variants which are typically fixed at conception, meaning that findings derived from this approach are more robust to confounding factors and reverse causation than findings from conventional observational epidemiological studies.

Recent findings emerging from the literature suggest that types of selection bias can hinder MR investigations, including survival bias which may distort findings when an outcome is measured in a nonrandom subset of the population who have survived long enough to be recruited into a study [3]. In this short communication, we propose the use of parental disease history data to help alleviate this source of bias in MR studies, given that the parents of individuals who have been diagnosed with a given disease will be considered a case regardless of their age at death. Furthermore, a recent study reported comparable results using case definitions based on family disease history in the UK Biobank (UKB) as when defining cases based on combined hospital records and questionnaire data, as well as increased statistical power for certain endpoints when using family history information [4].

**Fig. 1:**
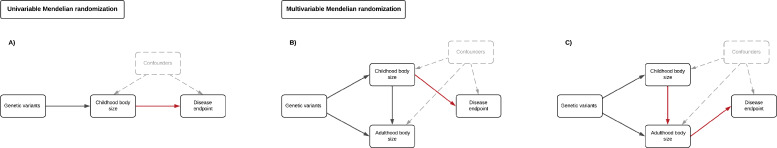
Schematic representation of applying (**A**) Univariable Mendelian randomization to estimate the ‘total effect’ of childhood body size on disease risk and and multivariable Mendelian randomization to separately estimates the (**B**) ‘direct effect’ and (**C**) ‘indirect effect’ of childhood body size on disease risk whilst accounting for the effect of adulthood body size (known as ‘lifecourse Mendelian randomization’). For example, previous applications of this approach have suggested that childhood body size has a direct effect (**B**) on risk of type 1 diabetes [6], but an indirect effect (**C**) on risk of type 2 diabetes [7]. These findings can be interpreted as indicating that being overweight in childhood exerts an effect in early life on risk of type 1 diabetes, whereas its influence on risk of type 2 diabetes is likely attributed to a sustained effect of remaining overweight at later stages of the lifecourse. The red arrows represent thee causal pathway being evaluated in each scenario

As an exemplar to demonstrate the value of analysing disease outcome data from first-degree relatives, we have investigated the genetically predicted effects of childhood body size on 8 major disease endpoints recorded for the parents of participants in the UKB (Supplementary Table 1). In doing so, we exploit the predictable genetic association between generations as a proxy for genotype-outcome estimates in measured cases, previously referred to as ‘proxy-genotype Mendelian randomization’ [5]. Findings were initially evaluated with univariable MR (Fig. 1A) and subsequently using a multivariable framework to estimate the direct and indirect effects of childhood body size on disease endpoints whilst accounting for the effect of adulthood body size (referred to as ‘lifecourse MR’ [6](Fig. 1B, C)).

## Methods

### Childhood and adult body size instrumental variables

Genetic instruments for childhood and adult body size were derived from a large-scale GWAS in the UKB conducted previously [7]. Full details of the GWAS protocol can be found in **Supplementary Note**. Linkage disequilibrium (LD) clumping was applied to identify our instruments using parameters of P < 5 × 10^− 08^ and r^2^ < 0.001 based on a reference panel based on 10,000 unrelated participants of European descent from UKB [8]. The final sets of genetic instruments can be found in Supplementary Table 2. These instruments have been validated in three independent populations which demonstrate their capability to reliably separate measured body mass index from childhood and adult timepoints as discussed in **Supplementary Note**. Furthermore, a recent study has found that the childhood genetic instruments have a much stronger effect on DXA-derived fat mass in early life compared to DXA-derived lean mass [9].

### Genetic estimates of disease outcomes using data on first-degree relatives

Reported illnesses of mothers (field 20110) and fathers (field 20107) were recorded in the UKB study by the majority of participants (n = 492,986 for maternal history and n = 488,077 for paternal history). Amongst these endpoints were; bowel cancer, breast cancer (mothers only), diabetes, heart disease, high blood pressure, lung cancer, prostate cancer (fathers only) and stroke. All outcomes were coded as 0 = neither parent with reported disease, 1 = one parent with disease and 2 = both parents with disease, with the exception of breast cancer and prostate cancer which was encoded as binary outcomes depending on whether mothers or fathers respectively had reportedly had these diseases. These fields in the UKB study were for blood relatives only as adopted mothers and fathers had separate fields for reported disease history (fields 20112 and 20113). If participants were unsure about any answers they were encouraged to respond with ‘do not know’. A summary of final sample sizes can be found in Supplementary Table 1. GWAS were applied to these outcome variables using the same protocol found in **Supplementary Note** to derive estimates for subsequent MR analyses.

### Statistical analysis

#### Mendelian randomization

Univariable MR analyses were initially undertaken to systematically estimate the total effect of genetically predicted exposures on each parentally proxied disease endpoint in turn. This was firstly conducted using the inverse variance weighted (IVW) method, which takes the SNP-outcome estimates and regresses them on those for the SNP-exposure associations. We subsequently applied the weighted median and MR-Egger methods which are more robust to horizontal pleiotropy than the IVW approach [2].

We next conducted multivariable MR to estimate the direct and indirect effects of exposures on disease endpoints which provided evidence of an effect based on FDR < 5% from IVW univariable analyses. Multivariable MR involves obtaining estimates for all instruments on each exposure being evaluated, thus allowing each estimated effect to take into account the effect of all other exposures in the model. Although this approach has been conventionally applied to analyse different risk factors as exposures (where estimates are typically interpreted as ‘lifelong effects’), the novelty of analysing the same exposure measured at different timepoints throughout the lifecourse (e.g. at age 10 and age 55 as conducted here) can facilitate inference in a lifecourse epidemiology setting. All analyses in this study were undertaken using R (version 3.5.1).

## Results

**Fig. 2:**
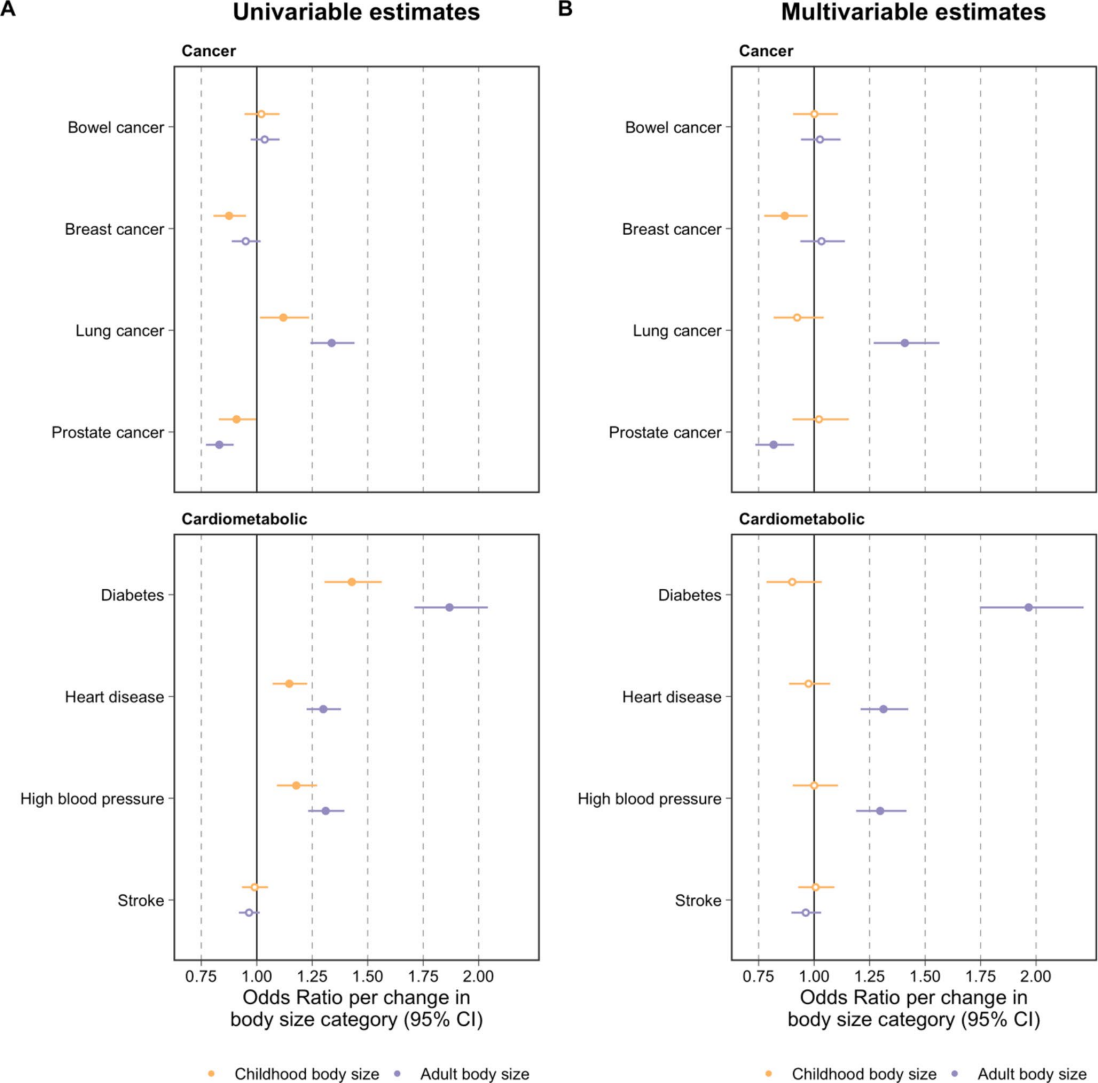
Univariable and multivariable Mendelian randomization estimates for childhood (yellow) and adult (purple) body size on risk of 8 major disease endpoints using parental history as proxy outcomes in the UK Biobank study

Applying univariable MR to parentally proxied outcomes provided evidence that childhood body size increases risk of disease endpoints such as heart disease (OR = 1.15, 95% CI = 1.07 to 1.23, P = 7.8 × 10^− 5^) and diabetes (OR = 1.43, 95% CI = 1.31 to 1.56, P = 9.4 × 10^− 15^) (Supplementary Table 3). However, effect estimates attenuated to be close to the null upon accounting for adulthood body size in a multivariable MR setting. This is in line with previous investigations, which suggest that childhood body size has no direct influence on these disease outcomes conditional on adulthood body size [7] (Supplementary Tables 4 & Fig. 2). Similarly, our results suggest that the genetically predicted effect of childhood body size on risk of parentally proxied lung cancer is likely attributed to individuals remaining overweight into adulthood (Fig. 2). We further investigated lifetime smoking as an additional exposure in our model, which we hypothesised likely resides along the causal pathway between body size and lung cancer risk as previously proposed [10]. Results supported this hypothesis as the effect of adulthood body size additionally attenuated upon accounting for the effect of lifetime smoking (OR = 1.11, 95% CI = 0.99 to 1.25, P = 0.08). Conversely, there was strong evidence of an effect of lifetime smoking on lung cancer risk whilst accounting for both childhood and adult body size (OR = 2.85, 95% CI = 2.42 to 3.35, P = 2.7 × 10^− 36^), suggesting that smoking mediates some of the effect of body size on lung cancer risk (**Supplementary Table 5**).

In contrast, we found evidence of a direct effect of childhood body size on risk of maternally proxied breast cancer (OR = 0.87, 95% CI = 0.78 to 0.97, P = 0.01) after accounting for the genetically predicted effect of adulthood body size as has been reported previously using findings from a large-scale consortium [7] (Fig. 2). We also found evidence of an indirect effect of childhood body size on paternally proxied prostate cancer risk via the pathway involving adulthood body size (OR = 0.82, 95% CI = 0.74 to 0.91, P = 2.1 × 10^− 4^). However, this finding requires further evaluation given that it has not been validated using data from the largest available prostate cancer consortium [7], which may potentially be explained by the paternal cases analysed in this study having a comparatively older age distribution compared to the consortium cases.

## Discussion

Our systematic evaluation of 8 major disease outcomes based on family disease history data using a lifecourse MR approach provides corroborating evidence into the long-term consequences of childhood body size. Such investigations would be challenging to undertake without the use of time-varying genetic variants harnessed as instrumental variables given the propensity of observational studies to be biased by confounding factors and reverse causation over the lifecourse. This study design using parental data also mitigates the influence of survival bias, which in particular emphasises the importance of developing insight into the aetiological relationship between childhood body size and breast cancer [11]. Furthermore, this approach may pave the way for mechanistic understanding into epidemiological relationships such as the effect of lifelong adiposity on lung cancer risk, which our findings suggest may be partly mediated by smoking.

There are however caveats to using disease history data in first-degree relatives with MR, such as the interpretation of effect estimates which in theory should be halved given that participants will on average share 50% of their DNA with individuals for whom outcomes occur [12]. For example, the multivariable MR estimate for adulthood body size on risk of diabetes using parental history data had a central effect estimate of OR = 1.97, which is approximately half the estimate reported previously using large-scale case control data (OR = 3.90) [6]. Supplementary Fig. 1 illustrates a side-by-side comparison of estimates derived in this study on parental endpoints with those from large-scale consortia.

Recent methodological developments to integrate individual-level case-control and family history data, such as the application of liability threshold modeling [13], may help improve the statistical power of downstream analyses such as MR. This is particularly attractive given that case numbers may be higher for disease outcomes in parents compared to individuals enrolled in a cohort, which has been exploited by genetic consortia for endpoints such as Alzheimer’s disease [14]. Future research is required to investigate the most appropriate manner to derive estimates using MR when outcomes are based on self- and parental reported endpoints. Lastly, these methods and the approach taken in this study rely on large-scale biobanks collecting data on family history data as pioneered by the UK Biobank. Where available these data provide a compelling source of evidence to triangulate findings from conventional MR investigations and therefore improve the robustness of investigations into lifecourse epidemiological relationships.

## Electronic supplementary material

Below is the link to the electronic supplementary material.

Supplementary Material 1

Supplementary Material 2

Supplementary Material 3

## Data Availability

All data on genetic instruments used in this study are located in Supplementary Tables 2 and the full genome-wide study summary statistics on parental outcomes will be made available via the GWAS catalog upon acceptance of publication.
