# Supplementary material for: Leveraging family history data to disentangle time-varying effects on disease risk using lifecourse mendelian randomization
Source: Eur J Epidemiol. 2023 May 8;38(7):765–9. doi: 10.1007/s10654-023-01001-8 (PMC10276123; doi:10.1007/s10654-023-01001-8)
Supplement: Supplementary file 2 — Supplementary Material 2 [file 10654_2023_1001_MOESM2_ESM.docx]

# **Supplementary Note**

## Genome-wide association study (GWAS) protocol

GWAS analyses were undertaken on all UK Biobank (UKB) participants of European descent based on K-means clustering (K = 4) after standard exclusions including withdrawn consent, mismatch between genetic and reported sex, and putative sex chromosome aneuploidy [1]. Single nucleotide polymorphisms (SNPs) associated with exposures and outcomes in this study were identified using the BOLT-LMM software [2]. BOLT-LMM uses a linear mixed model (LMM) to evaluate the association between genetic variants and phenotypic traits whilst accounting for population stratification and cryptic relatedness. Analyses were adjusted for age, sex, and a binary variable denoting the genotyping chip individuals were allocated to in UKBB (the UKBB Axiom array or the UK BiLEVE array). Further details on genotyping quality control, phasing, imputation, and association testing have been reported previously [3].

Although data from UKB were used in this study to obtain estimates for both our exposures and outcomes, using family reported data for outcomes should in theory reduce any potential overfitting as these are based on different individuals to those from which exposures estimates are based. Nonetheless, we evaluated this using the shiny application at <https://sb452.shinyapps.io/overlap> which suggested that type 1 error rates were low (i.e. < 0.05) based on the sample sizes, case numbers and instrument strength in our study. Forest plots in this study were generated using the R package ‘ggplot2’[4].

## Validation of childhood and adult body size instruments

Genetic instruments for childhood and adult body size have been previously validated using measured body mass index (BMI) data from 3 independent populations; the Avon Longitudinal Study of Parents and Children (ALSPAC) [5, 6], the Trøndelag Health (HUNT) study [7], and the Cardiovascular Risk in Young Finns Study [8]. Genetic correlation analyses have also demonstrated that the childhood body size genome-wide estimates are much more highly correlated with measured childhood obesity from an independent sample (rG = 0.85) compared to the adult measure (rG = 0.67). In contrast, results from the adult body size GWAS have been shown to be much more strongly correlated with measured BMI in adulthood (rG = 0.96) compared to the childhood measure (rG = 0.64). Conditional F-statistics generated for childhood (F = 13.6) and adult (F = 16.0) body size instruments suggest that weak instrument bias is unlikely to influence findings when using these sets of genetic variants in Mendelian randomization analyses.

# **Supplementary References**

[1] Anderson CA, Pettersson FH, Clarke GM, Cardon LR, Morris AP, Zondervan KT (2010) Data quality control in genetic case-control association studies. Nat Protoc 5(9): 1564-1573. 10.1038/nprot.2010.116

[2] Loh PR, Tucker G, Bulik-Sullivan BK, et al. (2015) Efficient Bayesian mixed-model analysis increases association power in large cohorts. Nat Genet 47(3): 284-290. 10.1038/ng.3190

[3] Bycroft C, Freeman C, Petkova D, et al. (2018) The UK Biobank resource with deep phenotyping and genomic data. Nature 562(7726): 203-209. 10.1038/s41586-018-0579-z

[4] Ginestet C (2011) ggplot2: Elegant Graphics for Data Analysis. J R Stat Soc a Stat 174: 245-245. DOI 10.1111/j.1467-985X.2010.00676_9.x

[5] Richardson TG, Sanderson E, Elsworth B, Tilling K, Davey Smith G (2020) Use of genetic variation to separate the effects of early and later life adiposity on disease risk: mendelian randomisation study. BMJ 369: m1203. 10.1136/bmj.m1203

[6] Waterfield S, Richardson TG, Smith GD, O’Keeffe LM, Bell JA (2022) Life stage-specific effects of genetic susceptibility to higher body size on body fat and lean mass: prospective cohort study. medRxiv: 2022.2004.2028.22274413. 10.1101/2022.04.28.22274413

[7] Brandkvist M, Bjorngaard JH, Odegard RA, et al. (2020) Separating the genetics of childhood and adult obesity: a validation study of genetic scores for body mass index in adolescence and adulthood in the HUNT Study. Hum Mol Genet. 10.1093/hmg/ddaa256

[8] Richardson TG, Mykkanen J, Pahkala K, et al. (2021) Evaluating the direct effects of childhood adiposity on adult systemic metabolism: a multivariable Mendelian randomization analysis. Int J Epidemiol. 10.1093/ije/dyab051
