# Supplementary material for: Leveraging family history data to disentangle time-varying effects on disease risk using lifecourse mendelian randomization
Source: Eur J Epidemiol. 2023 May 8;38(7):765–9. doi: 10.1007/s10654-023-01001-8 (PMC10276123; doi:10.1007/s10654-023-01001-8)
Supplement: Supplementary file 3 — Supplementary Material 3 [file 10654_2023_1001_MOESM3_ESM.docx]

**Figure S1 – Forest plots illustrating a comparison of Mendelian randomization estimates on 8 major disease outcomes using family history and large-scale case-control datasets**


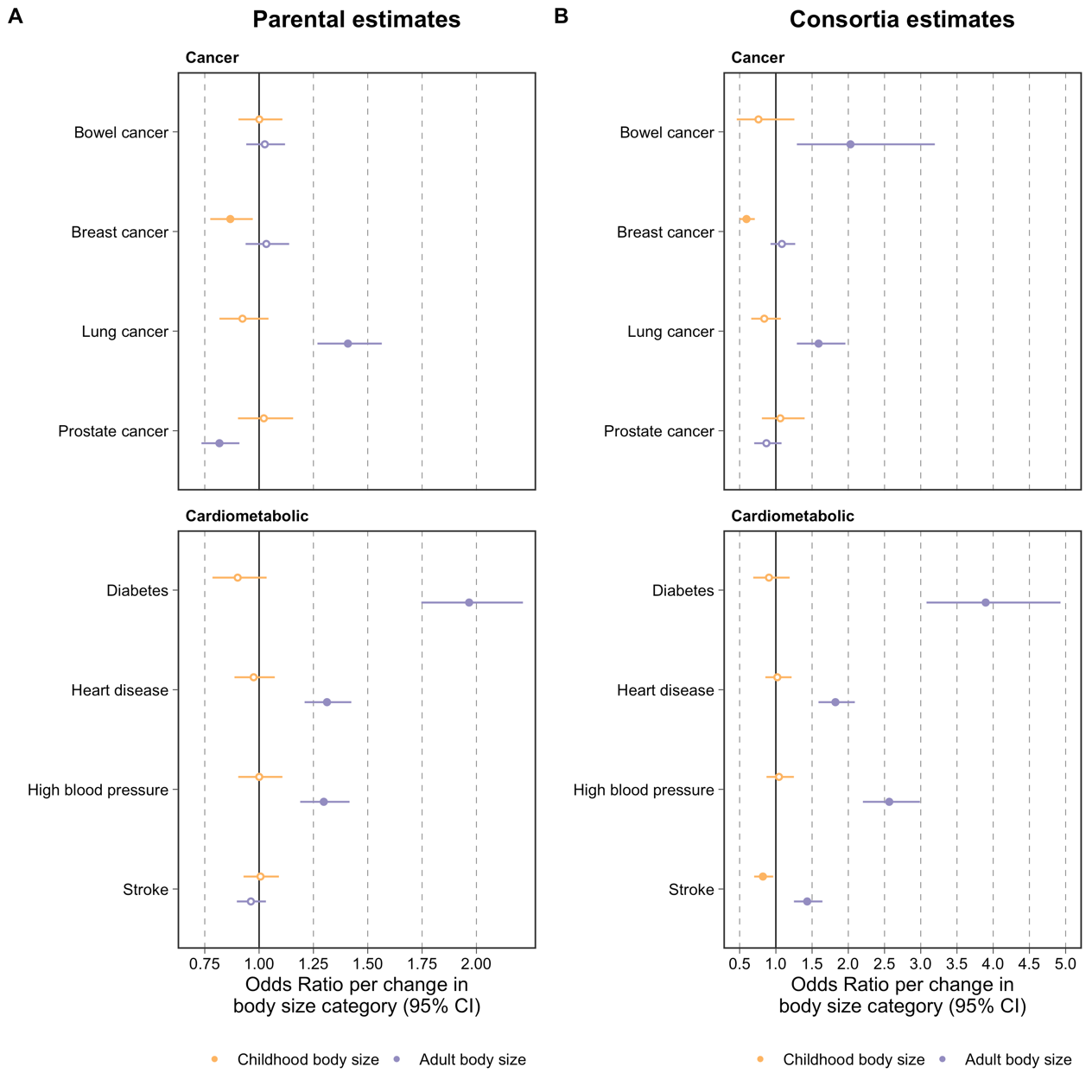


*Effect estimates for childhood (yellow) and adult (purple) body size on risk of 8 major disease endpoints derived using multivariable Mendelian randomization generated using A) family history data generated in this study and B) analyses on case-control outcomes provided by large-scale consortia. Consortia estimates for Heart disease, Diabetes, Prostate cancer and Breast cancer estimates taken from (Richardson et al., 2020), Bowel cancer and Lung cancer estimates taken from (Mariosa et al., 2022), High blood pressure estimates taken from (Power et al., 2021) and Stroke estimates were generated specifically for this figure using results provided by the MEGASTROKE consortium (Malik et al., 2018).*

Malik, R., Chauhan, G., Traylor, M., Sargurupremraj, M., Okada, Y., Mishra, A., Rutten-Jacobs, L., Giese, A. K., van der Laan, S. W., Gretarsdottir, S., Anderson, C. D., Chong, M., Adams, H. H. H., Ago, T., Almgren, P., Amouyel, P., Ay, H., Bartz, T. M., Benavente, O. R., Bevan, S., Boncoraglio, G. B., Brown, R. D., Jr., Butterworth, A. S., Carrera, C., Carty, C. L., Chasman, D. I., Chen, W. M., Cole, J. W., Correa, A., Cotlarciuc, I., Cruchaga, C., Danesh, J., de Bakker, P. I. W., DeStefano, A. L., den Hoed, M., Duan, Q., Engelter, S. T., Falcone, G. J., Gottesman, R. F., Grewal, R. P., Gudnason, V., Gustafsson, S., Haessler, J., Harris, T. B., Hassan, A., Havulinna, A. S., Heckbert, S. R., Holliday, E. G., Howard, G., Hsu, F. C., Hyacinth, H. I., Ikram, M. A., Ingelsson, E., Irvin, M. R., Jian, X., Jimenez-Conde, J., Johnson, J. A., Jukema, J. W., Kanai, M., Keene, K. L., Kissela, B. M., Kleindorfer, D. O., Kooperberg, C., Kubo, M., Lange, L. A., Langefeld, C. D., Langenberg, C., Launer, L. J., Lee, J. M., Lemmens, R., Leys, D., Lewis, C. M., Lin, W. Y., Lindgren, A. G., Lorentzen, E., Magnusson, P. K., Maguire, J., Manichaikul, A., McArdle, P. F., Meschia, J. F., Mitchell, B. D., Mosley, T. H., Nalls, M. A., Ninomiya, T., O'Donnell, M. J., Psaty, B. M., Pulit, S. L., Rannikmae, K., Reiner, A. P., Rexrode, K. M., Rice, K., Rich, S. S., Ridker, P. M., Rost, N. S., Rothwell, P. M., Rotter, J. I., Rundek, T., Sacco, R. L., Sakaue, S., Sale, M. M., et al. 2018. Multiancestry genome-wide association study of 520,000 subjects identifies 32 loci associated with stroke and stroke subtypes. *Nat Genet,* 50**,** 524-537.

Mariosa, D., Smith-Byrne, K., Richardson, T. G., Ferrari, P., Gunter, M. J., Papadimitriou, N., Murphy, N., Christakoudi, S., Tsilidis, K. K., Riboli, E., Muller, D., Purdue, M. P., Chanock, S. J., Hung, R. J., Amos, C. I., O'Mara, T. A., Amiano, P., Pasanisi, F., Rodriguez-Barranco, M., Krogh, V., Tjonneland, A., Halkjaer, J., Perez-Cornago, A., Chirlaque, M. D., Skeie, G., Rylander, C., Borch, K. B., Aune, D., Heath, A. K., Ward, H. A., Schulze, M., Bonet, C., Weiderpass, E., Smith, G. D., Brennan, P. & Johansson, M. 2022. Body size at different ages and risk of six cancers: a Mendelian randomization and prospective cohort study. *J Natl Cancer Inst*.

Power, G. M., Tyrrell, J., Frayling, T. M., Davey Smith, G. & Richardson, T. G. 2021. Mendelian Randomization Analyses Suggest Childhood Body Size Indirectly Influences End Points From Across the Cardiovascular Disease Spectrum Through Adult Body Size. *J Am Heart Assoc,* 10**,** e021503.

Richardson, T. G., Sanderson, E., Elsworth, B., Tilling, K. & Davey Smith, G. 2020. Use of genetic variation to separate the effects of early and later life adiposity on disease risk: mendelian randomisation study. *BMJ,* 369**,** m1203.
